# Nucleus-Targeting Phototherapy Nanodrugs for High-Effective Anti-Cancer Treatment

**DOI:** 10.3389/fphar.2022.905375

**Published:** 2022-05-11

**Authors:** Xingyu Long, Xiaojie Zhang, Qiaohui Chen, Min Liu, Yuting Xiang, Yuqi Yang, Zuoxiu Xiao, Jia Huang, Xiaoyuan Wang, Chong Liu, Yayun Nan, Qiong Huang

**Affiliations:** ^1^ Xiangya School of Pharmaceutical Sciences, Central South University, Changsha, China; ^2^ Department of Pharmacy, Xiangya Hospital, Central South University, Changsha, China; ^3^ National Clinical Research Center for Geriatric Disorders, Xiangya Hospital, Central South University, Changsha, China; ^4^ Hunan Provincial Key Laboratory of Cardiovascular Research, Xiangya School of Pharmaceutical Sciences, Central South University, Changsha, China; ^5^ Departments of Clinical Pharmacology and Pharmacy, Hunan Key Laboratory of Pharmacogenetics, and National Clinical Research Center for Geriatric Disorders, Xiangya Hospital, Central South University, Changsha, China; ^6^ Engineering Research Center of Applied Technology of Pharmacogenomics, Institute of Clinical Pharmacology, Ministry of Education, Central South University, Changsha, China; ^7^ Geriatric Medical Center, People’s Hospital of Ningxia Hui Autonomous Region, Yinchuan, China

**Keywords:** nucleus-targeting, photodynamic therapy, photothermal therapy, nanomaterials, subcellular organelle

## Abstract

DNA is always one of the most important targets for cancer therapy due to its leading role in the proliferation of cancer cells. Phototherapy kills cancer cells by generating reactive oxygen species (ROS) and local hyperthermia under light. It has attracted extensive interest in the clinical treatment of tumors because of many advantages such as non-invasiveness, high patient compliance, and low toxicity and side effects. However, the short ROS diffusion distance and limited thermal diffusion rate make it difficult for phototherapy to damage DNA deep in the nucleus. Therefore, nucleus-targeting phototherapy that can destroy DNAs *via in-situ* generation of ROS and high temperature can be a very effective strategy to address this bottleneck. Recently, some emerging nucleus-targeting phototherapy nanodrugs have demonstrated extremely effective anticancer effects. However, reviews in the field are still rarely reported. Here, we comprehensively summarized recent advances in nucleus-targeting phototherapy in recent years. We classified nucleus-targeting phototherapy into three categories based on the characteristics of these nucleus-targeting strategies. The first category is the passive targeting strategy, which mainly targets the nucleus by adjusting the physicochemical characteristics of phototherapy nanomedicines. The second category is to mediate the phototherapy nanodrugs into the nucleus by modifying functional groups that actively target the nucleus. The third category is to assist nanodrugs enter into the nucleus in a light-controlled way. Finally, we provided our insights and prospects for nucleus-targeting phototherapy nanodrugs. This minireview provides unique insights and valuable clues in the design of phototherapy nanodrugs and other nucleus-targeting drugs.

## Introduction

Currently, cancer has surpassed cardiovascular disease as the leading cause of death in many countries (Bray et al., 2018). In the United States, 1,918,030 new cancer cases and 609,360 cancer deaths are projected to occur in 2022 (Siegel et al., 2022). The uncontrolled proliferation of cancer cells is the most striking difference between cancer cells and normal cells (Hanahan, 2022). Destroying the DNA of cancer cells can very effectively inhibit the proliferation of cancer cells (Srinivas et al., 2019). Currently, a variety of anticancer drugs for DNA have been developed and occupied a prominent position in the treatment of cancer, many of which have become the first-line drugs, such as doxorubicin (DOX), cisplatin, and cyclophosphamide (Costoya et al., 2022; Jelinek and Zuchnicka, 2020; Li et al., 2021). Nonetheless, these drugs still have significant limitations in the treatment of cancer. DNA is deeply hidden in the nucleus of the cancer cell, and many drugs targeting DNA must pass through a series of barriers, such as the cytoplasmic membrane, lysosome, and nuclear membrane before entering the nucleus, making a large problem for drug delivery. In fact, only about 1–4% of cisplatin entered the nucleus after cellular internalization and even less (0.4%) of DOX entered the nucleus (Yu Cao et al., 2019; Lasorsa et al., 2019), which inevitably cause some serious side effects and poor prognosis.

**FIGURE 1:**
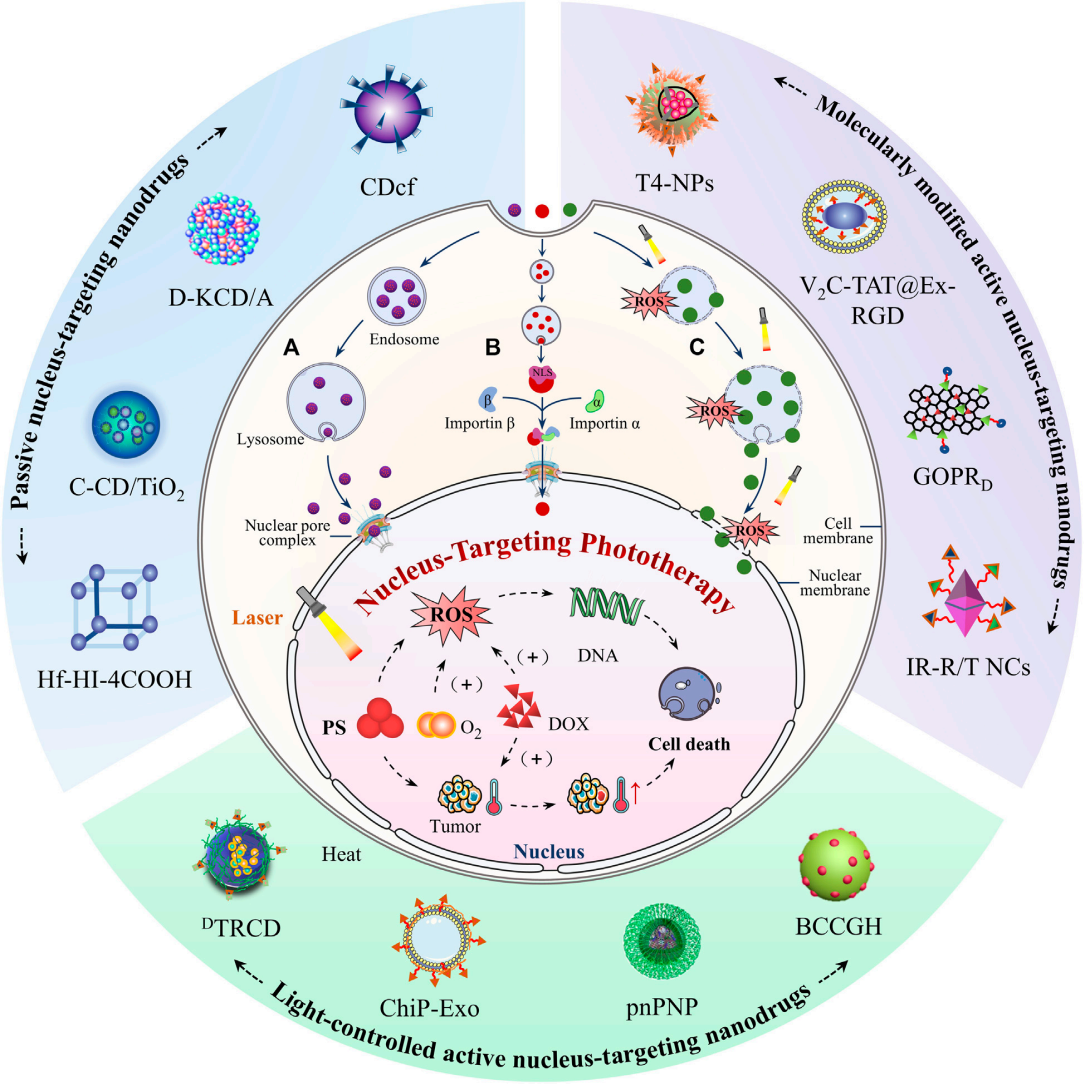
Schematic illustration of nucleus-targeting nanodrugs for enhancing PDT and PTT. According to the way nanodrugs targeting the nucleus, they are divided into two parts: passive nucleus-targeting nanodrugs **(A)** and active nucleus-targeting nanodrugs, which are further divided into molecularly modified active nucleus-targeting nanodrugs **(B)** and light-controlled active nucleus-targeting nanodrugs **(C)**. In **(A)**, nanodrugs that undergo size/hydrophilicity adjustment can passively penetrate the nucleus through NPCs after escaping from the endo/lysosome. In **(B)**, after being modified with the nucleus-targeting groups, nanodrugs can be recognized and then transferred into the nucleus by importin α/β through NPCs. As for **(C)**, ROS generated by nanodrugs under light irradiation will destroy a series of bio-membranes and facilitate the entry of nanodrugs into the nucleus. Nanodrugs accumulated in the nucleus generated a large amount of toxic ROS and local high temperature under light irradiation to destroy DNA and proteins in the nucleus and finally induce tumor cell death.

DNA is easily destroyed by reactive oxygen species (ROS) with strong nucleophilic activity because the bases of DNA are electrophilic (Srinivas et al., 2019). In addition, DNA is composed of a pair of single strands through Watson-Crick base pairing, and the unique structure is prone to high temperature (Chen et al., 2019; Pan et al., 2018). Phototherapy, mainly divided into photothermal therapy (PTT) and photodynamic therapy (PDT) (Gao et al., 2020), can efficiently damage DNA through ROS and local high temperature generated by photosensitizer (PS) under light irradiation (Chen et al., 2021; Wang et al., 2021). Phototherapy offers higher levels of patient compliance and medical safety thanks to its high selectivity and minimal invasiveness (Hailong Yang et al., 2021). Nevertheless, the “short-life” ROS and limited heat transfer rate greatly compromise the therapeutic effects of phototherapy (Deng et al., 2021; Chen et al., 2022; Zhao et al., 2022). Therefore, DNA-rich nucleus, as the central governor and an organelle that is most susceptible to ROS and high temperature is the ideal target for phototherapy (Jiang et al., 2020). Due to the central regulation of nucleus, nucleus-targeting phototherapy has better anticancer potential than other subcellular organelle-targeting phototherapy. However, the delivery of PS to the nucleus faces significant obstacles (Zhu et al., 2018; Rennick et al., 2021). The nucleus is wholly enclosed by a double nuclear membrane which hinders the connection between the nucleus and cytoplasm. But the nucleus is not completely isolated from the cytoplasm for the nuclear pore complexes (NPCs) embedded in the nuclear membrane mediate material exchange and information transfer. NPC is a hydrophilic channel with a nuclear pore of ∼39 nm (Zhen et al., 2021), through which hydrophilic small molecules can be diffused into the nucleus (Zimmerli et al., 2021). However, most biomacromolecules are difficult to enter the nucleus *via* passive diffusion due to their large size. Instead, they need to be recognized by nucleoporins and then enter or exit the nucleus (Shiyi Qin et al., 2021). From this perspective, PSs need to have both small size and hydrophilicity or be modified by the nuclear-driven molecule in order to enter the nucleus. As a matter of fact, most PS agents, such as porphyrin, chlorin e6 (Ce6), phthalocyanines, are hydrophobic and cannot penetrate the nuclear membrane to reach the interior of cancer cell nuclei (Chinna Ayya Swamy et al., 2020; Lo et al., 2020; Zhang et al., 2021). Fortunately, nanotechnology offers a huge opportunity to address the nucleus-targeting bottleneck of PSs (Xie et al., 2020). Recently, some emerging nucleus-targeting phototherapeutic nanodrugs have demonstrated far higher anti-tumor effects than traditional DNA-targeting drugs. Phototherapeutic nanodrugs can be passively targeted to the nucleus by changing surface properties and size because of the highly physicochemical flexibility of nanomaterials (Jiang et al., 2020; Xu et al., 2020). More importantly, active targeting of the nucleus can also be achieved by modifying nucleus-targeting groups such as cell-penetrating peptides [e.g., transactivator of transcription (TAT)] (Su et al., 2020; Tietz et al., 2022), the specific nuclear localization signal/sequence (NLS) peptide (Zelmer et al., 2020; Drescher et al., 2021), and DNA aptamers (Dong et al., 2018; Yang et al., 2018; Zeng et al., 2020) on phototherapeutic nanodrugs. Apart from this, ROS generated by phototherapeutic nanodrugs can also damage the membrane structure to increase permeability (Chen et al., 2021; Su et al., 2021; Chen et al., 2022). Subsequently, the phototherapeutic nanodrugs can remove barriers from lysosomal trapping and nuclear pore restriction, and finally enter the nucleus (Yuanyuan Qin et al., 2021). Currently, nucleus-targeting phototherapeutic nanodrugs have sparked extensive interest in the field of oncotherapy, but sadly, there were few relevant reviews. Herein, we reviewed the latest progress of nucleus-targeting phototherapeutic nanodrugs for cancer treatment based on improving PDT and PTT efficiency. We detailed the design strategies of nuclear passively and actively targeted nanodrugs and discussed future directions and application prospects of nucleus-targeting nanodrugs in tumor therapy (Figure 1; Table 1). This mini-review provides novel insights for tumor treatment and promotes the clinical application of PDT and PTT.

## Passive Nucleus-Targeting Nanodrugs

In order to facilitate the transport of macromolecules such as proteins and RNAs between the cytoplasm and the nucleus, the nuclear membrane maintains some pores that allow the passage of hydrophilic molecules smaller than 39 nm (Panagaki et al., 2021). Theoretically, nanodrugs with dimensions smaller than 39 nm and hydrophilicity have the potential for passive nuclear targeting (Fu et al., 2020). Carbon nanodots have been widely used in PDT, PTT, and fluorescence imaging in recent years due to their excellent optical properties. In addition, many carbon nanodots, which have small size and good hydrophilicity, are particularly suitable for passive targeting to the nucleus (Wareing et al., 2021). Recently, Nasrin et al. (2020) synthesized ultra-small conjugated carbon dots (CDcf) as two-photon active PSs for nucleus-targeting PDT. CDcf were obtained by solvothermal reaction of curcumin and folic acid at high temperature. Interestingly, CDcf retained part of the folic acid group, which enabled them to target folate receptor-positive oral cancer cells. CDcf entered the lysosome through folate receptor mediated endocytosis and formed a hybrid with the folate receptor. The hybrid released CDcf *via* lysosomal cleavage and then CDcf entered the nucleus due to its abundant hydrophilic amino groups and small size of 8 nm. Subsequently, the multi-conjugate system of CDcf mediated efficient photo-electron transfer to produce ROS and directly attacked DNA in the nucleus under irradiation with 780 nm laser. Benefiting from the effective nucleus-targeting ability, CDcf had much higher anticancer efficacy than CDc without nucleus-targeting ability. Similarly, Xu et al. found that N-doped carbon dots (Xu et al., 2021) and N/Se-doped carbon dots (Xu et al., 2020) with small size not only targeted the nucleus but also specifically bonded to RNA to further improve the treatment precision of carbon dots-mediated PDT. Nanoparticles with intrinsic nucleus-targeting ability can also serve as a drug-loading platform to deliver traditional PSs to the nucleus for high efficiency. For example, Lan Yang et al. (2021) reported an amino acid-based self-assembled nanocarrier with passive nucleus-targeting ability loaded with Ce6 and DOX (D-KCD/A) for the synergistic treatment of chemotherapy and PDT. D-KCD/A was prepared by azide crosslinkers and self-assemblies composed of dibenzocyclooctyne (DIBO)-functionalized lysine (D-K) and Ce6 by copper free click chemistry, followed by DOX loading. Because there were abundant hydrophobic regions, D-KCD/A had a high loading rate (17%) of Ce6, contributing to a higher tumor-killing efficiency. D-KCD/A was endocytosed and then entered the nucleus *via* a non-importin pathway thanks to the amphiphilic amino acid D-K. D-KCD/A exhibited a stronger antitumor effect than free Ce6 and D-KCD because of the synergistic effect of nuclear targeting and enhanced PDT.

In addition to PDT, passively nucleus-targeting PTT has also been developed. Recently, Jiang et al. (2020) developed a Hf-heptamethine indocyanine dye-based nanoscale coordination polymer (Hf-HI-4COOH) for nucleus-targeting PTT with low power density and temperature. Hf-HI-4COOH had a smaller size (36.7 nm) than NPCs and hydrophilicity derived from abundant uncoordinated carboxyl groups, which endowed it with good nuclear targeting ability. Up to 90% of Hf-HI-4COOH were delivered to the nucleus of 4T1 cells when incubated with Hf-HI-4COOH for 4 h. Hf-HI-4COOH significantly improved its photothermal conversion efficiency (39.51%) by the coordination between Hf and the heptamethine indocyanine dye, as demonstrated by a rapid temperature increase of ∼15°C within 5 min at the tumor site of tumor-bearing mice. Hf-HI-4COOH achieved effective tumor-killing effects while avoiding damage to the skin and normal organs under low power density (0.3 W/cm^2^) laser irradiation due to their high photothermal conversion efficiency and the intrinsic nucleus-targeting property. The tumor extracellular space is slightly acidic (pH = 6.5–6.8) because the tumor microenvironment is rich in lactate (Zhen et al., 2021; Huang et al., 2022; Zhu et al., 2022). To further improve the targeting of cancer therapy, Phuong et al. (2020) developed a slightly acidic environment-responsive carbon dot/TiO_2_ nanocomposites for nucleus-targeting PTT. Zwitterionic functionalized-carbon dots (Z-CDs) were attached to the surface of TiO_2_ nanocomposites *via* borolipid bonds. Under tumor slightly acidic conditions and visible light irradiation, the cleavage of the borolipid bonds resulted in the release of Z-CD from TiO_2_ nanoparticles, and Z-CD was internalized into tumor cells. The sulfobetaine moieties in Z-CD was protonized to lead to excellent hydrophilicity and small size in tumor microenvironment (23.5 nm in pH6.8). Benefiting from this, Z-CD efficiently entered the nucleus from the cytoplasm. The CD/TiO_2_ nanocomposites significantly upregulated the pro-apoptotic markers such as P53 and BAX in tumor cells and efficiently ablated tumors after being injected into the tumor and irradiated with Near Infrared (NIR).

## Active Nucleus-Targeting Nanodrugs

The nuclear passive targeting strategy has strict requirements on the size range and surface properties of nanodrugs, which limits the adaptability of this strategy. In addition, most strategies for passively targeting the nucleus rely on the passive diffusion of nanodrugs into the nucleus. Therefore, the delivery efficiency is relatively low and is negatively correlated with the particle size of nanodrugs. To achieve more effective nuclear targeting, many efficient active targeting strategies are currently developed for nanodrug-based phototherapy. These strategies can be divided into two categories. First, modifying the surface of nanodrugs with nucleus-targeting groups endows the nanodrugs with nucleus-targeting ability. Second, targeting the nucleus by light-controlled destruction of the nuclear membrane *via* the PDT or promotion of nanodrugs’ diffusion *via* PTT effects.

### Modification of Nucleus-Targeting Groups

Nucleus-targeting groups mainly include cell-penetrating peptides (TAT peptides, cyclic R10 peptides), NLS, and DNA aptamers. TAT, as the first discovered cell-penetrating peptide, is a highly cationic peptide with the sequence YGRKKRRQRRR derived from human immunodeficiency virus-1 (HIV-1). TATs have been demonstrated to transfer nanodrugs into the perinuclear region by binding to the import receptors importin α and then translocating through the nuclear pore *via* importin β (karyopherin) (Gao et al., 2019; Wan et al., 2020; Ziyang Cao et al., 2019). Cyclic R10 peptide (cR10), a kind of cyclic cell-penetrating peptide, has higher nucleus-targeting capabilities than both linear TAT and cyclic TAT peptides (Tu et al., 2020). NLS is an amino acid sequence that consists of one or more short sequences of positively charged lysine or arginine (Florio et al., 2022). The mechanism of NLS entry into the nucleus is the same as TAT but NLS does not mediate non-specific membrane penetration and has fewer side effects (Smith et al., 2018; Yue Tan et al., 2021). Histone, as a functional protein that constitutes chromatin, contains nuclear localization sequences that mediate nuclear targeting (Guo et al., 2022). Except for aptamers (Yang et al., 2018), these nucleus-targeting polypeptides and proteins have strong positive charges. Directly modifying these polypeptides and proteins on the surface leads to not only high toxicity but also instability in the blood circulation system because of the highly positive charge of nanodrugs. A general strategy is to modify the surface of these nucleus-targeting polypeptides and proteins based nanodrugs with long-chain polyethylene glycol (PEG) (Li et al., 2018; Wang et al., 2019), or coat a layer of exosomes to increase their biocompatibility (Ziyang Cao et al., 2019). More importantly, the abundant amino groups, hydroxyl groups and sulfhydryl groups in these peptides or proteins make them amenable to modification to mask their positive charge or be functionalized for tumor environmentally responsive activation. For instance, Zhang et al. (2021) developed nucleus-targeting nanodrugs (T4-NPs) cleaved in the lysosome for PDT. T4-NPs were prepared by co-assembly of SA-TAT (succinic anhydride-modified TAT)-PEG (poly (ethylene glycol))-PLA [poly (lactic acid)] and PEG-PLA followed by loading of TPE-TTMN-TPA (a new kind of photosensitizer). Benefiting from the charge masking of TAT by SA modifying, the surface potential of T4-NPs was negative, which made T4-NPs stable in the blood circulation system *in vivo*. When T4-NPs were endocytosed into the lysosomes of cancer cells, the acidic environment of the lysosome (pH = 5) prompted the cleavage of SA from the TAT of T4-NPs to lead a charge reversal (from −15.5 mV to +6.72 mV) and triggered TAT exposure to mediate T4-NPs’ entry into the nucleus. T4 NPs had highly efficient nuclear targeting and long nuclear retention (∼6 h), and the TPE-TTMN-TPA aggregated in the nuclei of tumor cells and generated a large amount of ROS under light irradiation, which significantly inhibited the proliferation of tumor cells and angiogenesis (anti-tumor rate of 78%).

Very recently, Tu et al. (2020) developed a graphene oxide (GO)-cR10 nuclear translocation nanoplatform loaded with DOX (GOPR_D_) for the synergistic antitumor effects of chemotherapy and PTT. GO contained abundant carboxyl groups with favorable photothermal property and were easily modified with hyperbranched polyglycerol (hPG) and cR10. In this case, the hPG was modified to improve the biocompatibility of GOPR_D_. The cR10 effectively promoted the internalization (ten-fold increase in absorption efficiency) and accumulation of GOPR_D_ in the nucleus. Under NIR light irradiation, GOPR_D_ exerted a high photothermal effect and effectively killed multi-drug-resistant HeLa cells. As expected, GOPR_D_ treatment demonstrated stronger anti-tumor activity than the free DOX group and non-nucleus-targeting group.

### Light-Controlled Nuclear Delivery

Many phototherapeutic nanodrugs are usually trapped in endo/lysosomes and are difficult to escape from them (Cheng et al., 2019a; Cheng et al., 2019b). Some specially designed nanodrugs can escape from endo/lysosomes through the proton sponge effect or charge reversal effect to better transport to nuclear. However, they are still likely to be blocked outside the nuclear membrane due to their large size. A strategy for efficient nuclear transport is to destroy endo/lysosomal and nuclear membranes by phototherapy-generated ROS or high temperature (Zhu et al., 2018; Yuanyuan Qin et al., 2021). For instance, Ziyang Cao et al. (2019) reported a PDT-driven nucleus-targeting PDT-nanodrugs (^D^TRCD) for cancer therapy. ^D^TRCD was prepared by self-assembly of TAT-modified ROS-responsive polymers followed by loading of Ce6 and DOX. Despite the presence of the nucleus-targeting group TAT peptide, ^D^TRCD cannot enter the nucleus and can only stay around the nucleus because of the large size of ^D^TRCD (40–200 nm). The toxic ROS generated by Ce6 directly destroyed the nuclear membrane integrity and the ROS-sensitive thiokel bonds in ^D^TRCD under light irradiation, which further promoted the intranuclear accumulation of Ce6 and DOX. As a result, ^D^TRCD treatment plus irradiation achieved the most effective tumor volume reduction compared to the free drug group and single sensitive nanomaterials group. In addition to PDT, PTT also mediates the diffusion of nanodrugs into the nucleus when they are near the nucleus. Recently, Hua et al. (2018) synthesized novel multifunctional nanodots (BCCGH) by mixing bovine serum albumin (BSA), carbon dots, metal ions (Cu^2+^ and Gd^3+^), and 2-(1-hexyloxyethyl)-2-devinyl pyropheophorbide-α (HPPH, a photosensitizer). BCCGH largely escaped the lysosomal entrapment due to the BSA surface and appropriate size/charge of the nanodots and were distributed mainly around the nucleus-endoplasmic reticulum. BCCGH had a very high photothermal conversion efficiency (68.4%). And high temperature promoted the further diffusion of BCCGH into the nucleus under mild NIR laser irradiation. The BCCGH targeted to the nucleus efficiently killed about 90% of cancer cells *via* ROS under laser light.

## Conclusion and Prospect

Nucleus-targeting phototherapy drugs can effectively solve the problem caused by the long distance between phototherapy drugs and their target DNA. Therefore, these emerging nucleus-targeting phototherapy nanodrugs have attracted a very wide range of research interests in the field of cancer therapy over the past several years. Herein, we systematically summarize recent advances in nucleus-targeting phototherapy nanodrugs for enhanced antitumor efficacy. To achieve satisfactory nucleus-targeting phototherapy, nanodrugs need to break through multiple hurdles (Zhu et al., 2018; Yu Cao et al., 2019): 1) effective tumor tissue-specific accumulation; 2) high efficiency of cellular internalization; 3) excellent endo/lysosomal escape ability; 4) fully perinuclear or intranuclear translocation, 5) efficient photo-thermal/photochemical energy conversion. Most of these well-designed active/passive nucleus-targeting nanodrugs can overcome these barriers to demonstrate excellent cancer therapeutic efficacy through irreversible and catastrophic thermal or oxidative damage to the nucleus. Despite the substantial advances gained, there are still various obstacles to overcome in furthering the clinical application of nucleus-targeting phototherapy nanodrugs. First, nanodrugs generally require a smaller size to achieve better nuclear targeting, especially for passively nucleus-targeting nanodrugs. However, the too small particle size of nanodrugs leads to the easy excretion of nanodrugs in the blood circulatory system through the kidneys or leakage into other normal tissues (Wei Tan et al., 2021). Second, many actively targeted phototherapeutic nanodrugs require additional modifications to maintain stability in the blood circulation system, which requires very sophisticated designs of nanodrugs to ensure exposure of active nucleus-targeting functional groups at appropriate sites group. Many sophisticated active-targeted nanodrugs are complicated to prepare and have many components, which will lead to a big bottleneck for the subsequent translation from laboratory to clinical. Third, the hypoxic environment at the tumor site can compromise the efficacy of nucleus-targeting nanodrugs PDT-based therapy (Zeng et al., 2020). Oxygen-independent PDT may solve the problems effectively. Fourth, phototherapy generally uses the near-infrared laser as the excitation light source. Although its tissue penetration has been greatly improved compared with visible light, it is still not suitable for many deep tissue tumors (Hailong Yang et al., 2021). To summarize, more efforts are still needed to improve the PDT and PTT effects provided by nucleus-targeting nanodrugs. Nevertheless, nucleus-targeting phototherapy nanodrugs remain intriguing research frontiers in the field of cancer treatment. It is foreseeable that the innovative design of nanodrugs will strongly promote the clinical translation of nucleus-targeting PDT and PTT.

**TABLE 1:** Nucleus-targeting nanodrugs for enhancing PDT and PTT.

Category	Nucleus-targeting strategies	Nanomaterials	Nucleus-targeting groups	Size (nm)	Therapy
Passive Nucleus-targeting nanodrugs	Size/hydrophilicity adjustment	CDcf Nasrin et al. (2020)	—	8	PDT
		I-CDs @FA Xu et al. (2021)		4.8(I-CDs)	PDT
		Se/N-CDs Xu et al. (2020)		3.6 ± 0.6	PDT
		D-KCD/A Hailong Yang et al. (2021)		∼10	PDT
		Hf-HI-4COOH Jiang et al. (2020)		30	PTT
		C-CD/TiO_2_ Phuong et al. (2020)		∼25 (Z-CD)	PTT
Active Nucleus-targeting nanodrugs	Modification of nucleus-targeting groups	TPE-TTMN-TPA NPs (T4-NPs) Zhang et al. (2021)	TAT	50–70	PDT
		Pd-TAT Gao et al. (2019)	TAT	19.7 ± 1.9	PTT
		TID NPs Wan et al. (2020)	TAT	108	PTT/Chemotherapy (CT)
		Ir-R/T NCs Wang et al. (2019)	TAT	<5	PDT/Radio-therapy (RT)
		CuS@MSN-TAT-RGD Li et al. (2018)	TAT	∼40	PTT
		V_2_C-TAT@Ex-RGD Yu Cao et al. (2019)	TAT	71	PTT
		GOPR_D_ Tu et al. (2020)	CPPs	∼20	PTT/CT
		DIR825@histone Guo et al. (2022)	Histone	∼37.5	PDT/CT
		CACH-PEG Yang et al. (2018)	AS1411	∼65 (pH6.5) ∼70 (pH7.4)	PDT/CT
	Light-controlled nuclear delivery	^D^TRCD Ziyang Cao et al. (2019)	TAT	63.6 ± 9.6	PDT/CT
		BCCGH Hua et al. (2018)	—	∼7.9	PDT/PTT
		pnPNP Cheng et al. (2019a)	NLS	157.6 ± 2.5	PDT
		ChiP-Exo Cheng et al. (2019b)	NLS	132.6	PDT
		PPR NPs Zhu et al. (2018)	—	∼75	PDT
			Ce6-C_18_-PEG/^125^I-Cur Shiyi Qin et al. (2021)	—	10–30	PDT
